# Balancing Sodium and Potassium: Estimates of Intake in a New Zealand Adult Population Sample

**DOI:** 10.3390/nu7115439

**Published:** 2015-10-28

**Authors:** Rachael McLean, Julia Edmonds, Sheila Williams, Jim Mann, Sheila Skeaff

**Affiliations:** 1 Department of Human Nutrition, University of Otago, P.O. Box 56, Dunedin 9054, New Zealand; kiwijulia@yahoo.co.uk (J.E.); jim.mann@otago.ac.nz (J.M.); sheila.skeaff@otago.ac.nz (S.S.); 2 Department of Preventive and Social Medicine, Dunedin School of Medicine, University of Otago, P.O. Box 56, Dunedin 9054, New Zealand; sheila.williams@otago.ac.nz; 3 Nutrition Society of New Zealand, PO Box 2039, Whanganui 4543, New Zealand

**Keywords:** sodium, potassium, dietary, adult, New Zealand

## Abstract

Dietary intakes of sodium and potassium are important determinants of blood pressure. We assessed sodium and potassium intake in a cross-sectional survey which included a random sample of New Zealand Adults aged 18 to 64 years from two New Zealand cities: Dunedin and Wellington. Participants completed a short questionnaire, had height, weight and blood pressure measured, and collected a 24 h urine sample. Mean 24 h sodium excretion was 3386 mg/day (95% CI 3221, 3551): 3865 mg/day for men and for 2934 mg/day women. Mean 24 h potassium excretion was 2738 mg/day (95% CI 2623, 2855): 3031 mg/day for men and 2436 mg/day for women. Mean sodium:potassium ratio was 1.32 (95% CI 1.26, 1.39); 1.39 for men and 1.26 for women. Sodium intake was higher among younger people, men, those with a higher BMI and higher potassium excretion. Potassium excretion was higher among older people, men and those with a higher sodium excretion. New Zealand adults have high sodium intakes and low potassium intakes compared to recommended levels. This is likely to adversely affect population blood pressure levels as well as incidence of cardiovascular disease. A comprehensive public health programme to reduce dietary sodium intake and increase intake of fruit and vegetables is warranted.

## 1. Introduction

A dietary pattern which is low in sodium and high in fresh fruit and vegetables (and therefore high in potassium) has been recommended for blood pressure lowering, and is well supported by numerous randomised controlled trials [1,2,3]. The World Health Organization (WHO) 2013–2020 Global Action Plan for the Prevention and Control on Noncommunicable Diseases identifies nine voluntary global targets including a 25% reduction in risk of cardiovascular disease and raised blood pressure, and a 30% reduction in mean population salt intake by 2025 [4]. WHO also recommends that adults consume a minimum of 400 g of fruit and vegetables per day (excluding starchy root vegetables such as potatoes) for the prevention of chronic disease [5]. A sodium: potassium intake ratio of approximately 1 has been recommended for optimal health [6,7], with higher ratios associated with elevated blood pressure and increased risk of cardiovascular disease [8]. WHO recommends a maximum sodium intake for adults of 2 g per day (equivalent to 5 g of salt) [7].

In New Zealand a number of previous population based surveys have shown that dietary potassium intakes are lower, and dietary sodium intakes are substantially above recommended levels. The most recent estimate of population potassium intake comes from 24 h diet recall used in the 2008/09 New Zealand Adult Nutrition Survey (2008/09NZANS). These results show the median usual daily intakes of potassium were 3449 mg/day for men and 2757 mg/day for women, with no significant differences by ethnicity or socio-economic status. These are below the recommended adequate intake (AI) [9]. The last reported data on sodium intakes in New Zealand are based on the collection of 24 h urine samples from healthy volunteers between 1993 and 1998 in two New Zealand provinces reporting a sodium intake of 3464 mg/day (3854 for men and 3122 for women) [10]. We aimed to update an assessment of sodium and potassium intake from 24 h urine samples with biochemical validation of completeness (the gold standard [11]) in a random sample of adults living in two main cities in New Zealand.

## 2. Experimental Section

This cross-sectional survey was conducted between February and November 2012. Adults aged between 18 and 64 years were identified from the New Zealand electoral roll (including both the general and Māori rolls) in two cities in New Zealand (Wellington (population = 191,000)) [12], and Dunedin (population = 119,000)) [13]. A random sample with an equal number of men and women in each of three age groups (18–24 years, 25–44 years, and 45–64 years) was drawn from the electoral roll. Participants were invited by post, and followed up a maximum of three times using postcards and telephone contact if required, all participants have signed an informed consent form. Ethical approval was obtained from the University of Otago Ethics Committee (reference 11/244).

We anticipated proportions participating of 50% in Dunedin and 35% in Wellington based on results of a previous similar study [14]. Because the proportion participating among 18–24 year old men was low, a snowball (word of mouth) technique was used to recruit further participants who were not family members of existing participants in this demographic group. Participants were eligible if they met the following criteria: no history of thyroid, heart, or kidney disease; not taking any thyroid medication or had not started diuretics in the previous 2 weeks. They attended a clinic session with a trained interviewer and completed a questionnaire, which included sociodemographic information (age, sex, ethnicity, level of income), and medication use. Ethnicity was self-identified and participants were categorised into three ethnic groups: Māori, Pacific, and New Zealand European and Others (NZEO).

Participants underwent standardised height, weight and blood pressure measurements. Blood pressure was measured using the HEM-7221 (M6 comfort, Omron Healthcare, Inc., Lake Forest, IL, USA) automated sphygmomanometer with an appropriately sized cuff [15]. Three blood pressure measurements were taken one minute apart, and the mean of the second and third readings were used for this analysis.

Twenty-four hour urine collections were obtained following a protocol developed by the Pan American Health Organization Regional Expert Group for Cardiovascular Disease Prevention [16]. Participants were given collection equipment (including a two and five litre container, a jug and funnel and information sheet) and instructions for completing a 24 h urine collection on a day most convenient to them. They were asked to record time of collection (including start and finish time) and whether they had missed any urine collections during the 24 h period. After collection, participants were instructed to contact a courier company and asked to place equipment and completed 24 h urine sample in pre-paid courier bags which were subsequently couriered to local clinics. At the clinic, the total volume of each 24 h urine collection was recorded, and two aliquots stored at −20 °C. Frozen urine samples from Wellington participants were couriered to Dunedin; all samples were stored at the Department of Human Nutrition, University of Otago, Dunedin at −20 °C until analysis. Urine samples were thawed at room temperature, vortexed, and analysed in batches in Department of Human Nutrition laboratories. Urinary sodium and potassium were determined on a Roche Hitachi Cobas C311 ISE unit biochemical analyser using an ion selective electrode. Urinary creatinine was determined by Jaffe reaction using alkaline picrate (Roche Hitachi Cobas C311 analyser). Completeness of 24 h urine collection was assessed in three ways: firstly participants were asked to report whether they had missed any urine collections, and results were not included if they reported missing two or more collections. Secondly, 24 h creatinine excretion was estimated using a formula developed by Joossens and Geboers [17] (estimated 24 h creatinine excretion (mg/day) = 24 × body weight (kg) for men and 21 × body weight for women) with participants excluded with an observed 24 h creatinine excretion less than 60% of the estimated value [17]. Thirdly, collections were excluded if they were less than 0.5 L in volume.

### Statistical Analysis

All statistical analyses were carried out using the statistical package STATA 11.1 (StataCorp LP. College Station, TX, USA). Both 24 h urinary excretion of sodium and potassium were used as estimates of daily dietary intake. Population means were estimated for men and women aged 18 to 64 years by weighting data for each respondent according to population estimates by age and sex from the New Zealand Census for 2012 [18]. The Stata survey command was used to estimate population means and confidence intervals [19]. Linear regression analysis was used to determine the effect of age, sex (male, female) and 24 h sodium excretion. As 24 h sodium excretion results are slightly positively skewed, the regression analysis was undertaken with both log-transformed and untransformed 24 h sodium excretion data.

## 3. Results

778 (57%) of the 1525 invited did not respond to any postal invitations, three had died and 149 invitation letters were returned indicating incorrect address. Of the 598 who responded, 305 people declined to participate, and 16 were ineligible, leaving 274 who agreed to participate in the study. Of these only 251 completed the study- a final proportion participating of 17% of those initially invited. A further 54 people recruited via snowball sampling complemented the sample, with 50 completing the study. Of those who completed the study two 24 h urine samples were excluded because they were less than 0.5 L (0.42 L and 0.49 L). No participants reported missing any collections, and none were excluded on the basis of inadequate creatinine excretion. The final sample included 299 participants.

Baseline characteristics of those included in the final analysis are presented in Table 1. 51% were women and 94% were of NZEO ethnicity. Six percent of participants (*n* = 18) reported that they were taking medication for high blood pressure, and of these only two reported taking diuretic medications (both thiazide diuretics). Mean (SD) urine volume was 1.99 L (0.95) with a range of 0.57 to 7.34 L; 2.08 (1.06) L for men and 1.91 (0.83) L for women. Results from the urinary analysis for sodium, potassium and creatinine are shown in Table 2. Mean 24 h sodium excretion was 3386 mg/day (95% CI 3221, 3551): 3865 mg/day for men and 2934 mg/day for women. Mean 24 h potassium excretion was 2738 mg/day (95% CI 2623, 2855): 3031 mg/day for men and 2436 mg/day for women. Excluding one outlier of 10,605 mg/day, the mean 24 h potassium excretion was 2702 mg/day (95% CI 2608, 2816). Mean sodium:potassium ratio was 1.32 (95% CI 1.26, 1.39); 1.39 for men and 1.26 for women. Means weighted to reflect population proportions by age and sex are: for sodium excretion 3377 mg/day (95% CI 3202, 3551), potassium excretion 2744 mg/day (95% CI 2632, 2855) and sodium:potassium ratio 1.3 (95% CI 1.2, 1.4). Mean 24 h creatinine excretion was 1422 mg/day: 1710 mg/day for men and for 1151 mg/day women. The correlation between 24 h sodium excretion and 24 h potassium excretion was 0.36 (*p* < 0.001)

**Table 1:** Baseline Characteristics of Participants.

Characteristics	Men (*N* = 145)	Women (*N* = 154)	Total (*N* = 299)
City of residence: (number (%))			
Dunedin	75 (52)	83 (54)	158 (53)
Wellington	70 (48)	71 (46)	141 (47)
Age (number (%))			
18–24 years	44 (30)	41 (26)	85 (28)
25–44 years	49 (34)	55 (36)	104 (35)
45–64 years	52 (36)	58 (38)	110 (37)
Ethnicity: (number (%))			
Māori	3 (2)	9 (6)	12 (4)
Pacific	2 (1)	5 (3)	7 (2)
NZEO	140 (97)	140 (91)	280 (94)
Income: (number (%))			
<50,000 NZD per year	74 (51)	100 (66)	174 (59)
>50,000 NZD per year	62 (43)	36 (24)	98 (33)
Did not respond	9 (6)	15 (10)	24 (8)
Body size: mean (sd)			
Height (cm)	177.2 (7.3)	164.3 (5.9)	170.5 (9.2)
Weight (kg)	83.3 (15.0)	72.6 (16.2)	77.8 (16.2)
BMI (kg/m^2^)	26.5 (4.4)	26.9 (5.8)	26.7 (5.2)
Blood pressure: mean (sd)			
Systolic (mmHg)	132 15)	120 (14)	126 (16)
Diastolic (mmHg)	78 (12)	76 (11)	77 (11)

NZEO: New Zealand European and Other; NZD: New Zealand Dollar; BMI: Body Mass Index.

**Table 2:** 24 h sodium, potassium, iodine and creatinine excretion: mean, standard deviation (sd) and 95% Confidence Interval (95% CI)

Group	Sodium (mg/day)		Potassium (mg/day) *		Sodium:Potassium Ratio *		Creatinine (mg/day)
	Mean (sd)	95% CI	Mean (sd)	95% CI	Mean (sd)	95% CI	
**Men:**							
18–24 years	3866 (1554)	3405, 4327	2766 (813)	2525, 3008	1.50 (0.67)	1.30, 1.69	1706 (431)
25–44 years	3795 (1503)	3372, 7217	3058 (957)	2786, 3329	1.29 (0.45)	1.16, 1.42	1703 (482)
45–64 years	3931 (1520)	3517, 4347	3085 (1050)	2798, 3371	1.38 (0.62)	1.21, 1.55	1719 (401)
Total men	3865 (1515)	3618, 4113	2979 (955)	2822, 3135	1.39 (0.59)	1.29, 1.48	1710 (436)
Weighted mean †	3854	3597, 4111	3005	2837, 3174	1.36	1.27, 1.46	N/A
**Women:**							
18–24 years	3017 (954)	2724, 3311	2193 (632)	1999, 2387	1.43 (0.46)	1.29, 1.57	1174 (232)
25–44 years	3035 (1241)	2706, 3365	2361 (839)	2138, 2583	1.37 (0.52)	1.23, 1.51	1188 (283)
45–64 years	2780 (1401)	2418, 3142	2751 (784)	2549, 2954	1.04 (0.44)	0.93, 1.16	1100 (350)
Total women	2934 (1235)	2738, 3130	2463 (798)	2337, 2590	1.26 (0.51)	1.18, 1.34	1151 (299)
Weighted mean ^†^	2926	2716, 3137	2497	2363, 2631	1.24	1.16, 1.33	N/A
Total men and women	3386 (1452)	3221, 3551	2712 (913)	2608, 2816	1.32 (0.55)	1.26, 1.39	1422 (465)

* excluding one outlier with potassium excretion of 10,605 mg/day; ^†^ weighted to reflect age and sex structure of the New Zealand population aged 18–64 years in 2012.

The majority (77%, *n* = 229)) of participants had a sodium excretion that exceeded the Upper Level of Intake of 2300 mg/day (85% of men and 69% of women). Only 23% (*n* = 69) of participants had an adequate intake of potassium (2800 mg/day for women and 3800 mg/day for men) (18% of men and 28% of women) according to current New Zealand Nutrient Reference Values [20].

Table 3 shows the results of multiple linear regression with 24 h sodium and 24 h potassium as outcome variables. In multiple linear regression, 24 h sodium excretion was significantly negatively associated with increasing age, and was positively associated with male sex, body weight and potassium excretion. There was no significant association between 24 h sodium excretion and systolic blood pressure when controlling for age, sex, body weight and 24 h potassium excretion. As 24 h sodium excretion results are positively skewed, the multivariate analysis was repeated using log-transformed 24 h sodium excretion data (results not shown). As this did not substantially change the substance of the results, those using un-transformed data are shown here.

**Table 3:** Variables associated with 24 h urinary sodium and potassium excretion (multiple linear regression analyses) *.

Models	β Coefficient	95% CI	*p*
**Association with 24 h sodium excretion (mg/day)**			
Age	−16	−27, −6	0.003
Male sex	371	702, 39	<0.028
Body weight (kg)	23	13,32	<0.001
Potassium excretion (mg/day)	0.53	0.36, 0.69	<0.001
Systolic Blood Pressure	2	−8, 13	0.663
**Association with 24 h potassium excretion (mg/day)**			
Age	11	4, 17	0.002
Male sex	279	65, 494	0.011
Body weight (kg)	3	−3, 9	0.369
Sodium excretion (mg/day)	0.22	0.15, 0.29	<0.001
Systolic Blood Pressure	1	−6, 8	0.768

* excluding one outlier with potassium excretion of 10,605 mg/day.

Potassium excretion was positively associated with age and sodium excretion, and male sex. There was no significant association between 24 h potassium excretion and systolic blood pressure, when controlling for age, sex, body weight and 24 h sodium excretion.

## 4. Discussion

We found that this sample of New Zealand adults aged 18–64 had low potassium intakes and high sodium intakes compared with current New Zealand and international guidelines. This is reflected in the unfavourable sodium:potassium ratio which is above 1.0 for all groups, except women aged 45–64 years. Sodium intake was higher among younger people, men, those with a higher BMI and higher potassium excretion. Potassium excretion was higher among older people, men and those with a higher sodium excretion. No association was demonstrated between sodium or potassium excretion and systolic blood pressure. These results indicate that improvements can be made to the dietary intake of New Zealand adults in order to reduce risk of cardiovascular disease, a leading cause of morbidity and mortality.

Our results also showed inadequate potassium intakes overall, and sodium:potassium ratios above 1.0 in all sub-groups other than women 45–64 years. This is consistent with potassium intake estimates from the 2008/09NZANS, which showed inadequate intakes from 24 h diet recall, (median usual daily intake of potassium of 3449 mg for males and 2757 mg for females, below the AI of 3800 mg/day for men and 2800 mg/day for women). The 2008/09NZANS results show that only 59% of males and 72% of females reported eating three or more servings of vegetables a day, and only 55% of males and 66% of females reported eating two or more servings of fruit each day [9], which is likely to have contributed to the low observed potassium intakes in the 2008/09 NZANS as well as in our study.

Young adults, men and those with a higher BMI were more likely to have a high sodium intake. Sodium intake is highly correlated with energy intake [21] as it is highly prevalent in most processed foods, and this may be the main reason for these associations. Furthermore, young adults may be more likely to consume sodium dense foods such as takeaways and highly processed snacks than older adults [22]. In this survey young adults had a lower potassium intake suggesting a lower intake of fruit and vegetables than older adults. However, overall there was a positive correlation (*r* = 0.36) between sodium and potassium intake, and a positive association demonstrated on regression analysis, indicating that those with a higher sodium intake also had a higher potassium intake. This is likely to reflect an association with both sodium and potassium and energy intake, although this could not be assessed in this study. The lack of association demonstrated between systolic blood pressure and either sodium or potassium intake is not surprising, as this was a cross-sectional survey and not designed or powered to investigate this relationship.

Subject to the limitations inherent in studies of this type, the results of this survey suggest that New Zealand adults’ sodium intake has remained remarkably stable since the late 1970s, despite decades of increasing evidence of the harmful effects of elevated sodium reduction. A 1975 survey of 1200 adults in a small South Island town (the Milton Survey) using 24 h urine collections, showed the average sodium excretion for men was 3979 mg/day and for women was 3197 mg/day [23]. A subsequent 24 h urinary assessment in two New Zealand provinces between 1993 and 1998 showed a mean sodium excretion of 3854 mg/day for men and 3122 mg/day for women [10]. Although it is disappointing that sodium intakes appear not to have decreased over this time, this pattern is consistent with estimates of sodium intakes in the United States and other countries over a similar time period [24,25]. It is also unsurprising for a number of reasons. Sodium is ubiquitous in the food supply. Estimates show that only about 10% of sodium intake from a Western-style diet is from salt added in the home in cooking or at the table, with around three quarters coming from sodium present in processed foods [26]. There is limited ability for individuals to control their own sodium intake in the absence of a concerted public health programme of reformulation of processed foods, especially as they find it difficult to interpret nutrition labels in this context [27]. Although some efforts are being made to lower population sodium intake, New Zealand has not implemented a comprehensive government led strategy such as that recently modelled in the United Kingdom. In New Zealand sodium reduction initiatives are currently led by the Heart Foundation in a programme called HeartSAFE: an industry led initiative to reduce the sodium content in bread, breakfast cereals, processed meat, savoury pies, soups, cheese, savoury snacks, cooking sauces and edible oil spreads [28]. This falls short of initiatives in other countries, such as South Africa (where a series of mandatory sodium concentration targets have recently been introduced for a range of processed foods) [29], and in the United Kingdom. The United Kingdom salt reduction programme was initiated in 2003 by the Food Standards Agency, and has included a consumer education programme, improvements in food labelling, as well as collaboration with food industry partners to achieve sodium reduction across a wide range of food products based on specific sodium concentration targets [30]. The implementation of the United Kingdom programme has been associated with a significant reduction in population sodium intake of 15%: from around 3800 mg/day in 2003 to around 3200 mg/day in 2011. This estimate is based on 24 h urinary assessment in a population sample with a similar age range to that of our study. The reduction in sodium intake has been accompanied by a reduction in population blood pressure levels as well as reductions in mortality from ischaemic heart disease and stroke [30,31].

### Strengths and Limitations

This is the first New Zealand survey estimating both sodium and potassium intakes using the gold standard 24 h urinary assessment in a randomly selected sample. A number of limitations exist however. Firstly, while we used the electoral roll to select a population sample, we only selected people from two cities: Wellington and Dunedin, which may not be generalisable to the whole New Zealand population. The proportion participating was relatively low at only 17%, which may have biased results, however it is difficult to estimate how such a bias may have operated. There is some limited evidence that sodium intakes do not appear to be strongly associated with propensities to take part in surveys. In Australia, for example, a population based survey using 24 h urine collection had only 16% participating, although results were not significantly different between those invited participants and healthy volunteer recruits with respect to sodium excretion [32]. There was also no significant difference between participants recruited via the electoral roll, and those who volunteered through snowball sampling in our study. Compared with 2006 New Zealand Census data for each city [12,13], there was a higher proportion of participants of NZEO ethnicity and with incomes greater than $50,000 than the general population. It is possible therefore that based on income and the low proportion participating, those who agreed to participate were more likely to consume a more healthy diet (higher in potassium and lower in sodium) overall. Participants with a prior diagnosis of hypertension (*n* = 18) may be more likely to have been advised to restrict sodium intake, however hypertension is a common condition and the inclusion of these people is reflective of the population in this age range. Although 24-h urine is the gold-standard method of sodium and potassium intake, this is likely to be an under-estimate of total intake since 90% of sodium consumed is excreted in the urine. Furthermore, both under-and over collection of 24 h urine have been reported in other surveys [33]. While we used several widely accepted methods to exclude samples that were inadequate, under-collection of urine over the 24 h period cannot be excluded. Two participants reported being on long-term thiazide diuretics, however we believe that this is unlikely to have substantially altered sodium and potassium excretion results in this study.

## 5. Conclusions

New Zealand adults have high sodium intakes and low potassium intakes compared to recommended levels. This is likely to adversely affect population blood pressure levels as well as incidence of ischaemic heart disease and stroke. If New Zealand is to comply with the WHO recommendation to reduce sodium intake by 30% by 2025, a comprehensive public health programme to reduce dietary sodium intake and increase intake of fruit and vegetables, thereby increasing potassium intake, is warranted.
