# Correction: McLean, R., Edmonds, J., Williams, S., Mann, J., Skeaff, S. Balancing Sodium and Potassium: Estimates of Intake in a New Zealand Adult Population Sample. *Nutrients* 2015, *7*(11), 8930–8938

**DOI:** 10.3390/nu8020065

**Published:** 2016-01-26

**Authors:** Rachael McLean, Julia Edmonds, Sheila Williams, Jim Mann, Sheila Skeaff

**Affiliations:** 1 Department of Human Nutrition, University of Otago, P.O. Box 56, Dunedin 9054, New Zealand; kiwijulia@yahoo.co.uk (J.E.); jim.mann@otago.ac.nz (J.M.); sheila.skeaff@otago.ac.nz (S.S.); 2 Department of Preventive and Social Medicine, Dunedin School of Medicine, University of Otago, P.O. Box 56, Dunedin 9054, New Zealand; sheila.williams@otago.ac.nz

We would like to submit the following as a correction to our recently published paper. We calculated sodium-potassium ratios and compared them to an optimal sodium–potassium ratio recommended by WHO. The WHO optimal sodium–potassium ratio of 1.0 referred to in our paper is a molar ratio [1], whereas we have calculated the ratio (Table 2) using intakes expressed in mgs. There is some confusion in the literature on this issue, with some authors reporting the ratio using units of mg/day [2]. However, we accept that it is conventional, and therefore more useful from a public health standpoint, to report a molar ratio.

We include here a revised version of Table 2 that contains the sodium–potassium molar ratio. Interpretation of these results is largely the same, *viz*. that there is an unfavourable sodium–potassium ratio above 1 for all groups, rather than for all groups except women aged 45–64 years, which was demonstrated in the previous analysis. We would like to apologise for any confusion caused by this error in our paper.

**Table 2:** The 24 h sodium, potassium and creatinine excretion: mean, standard deviation (sd) and 95% Confidence Interval (95% CI).

Group	Sodium (mg/day)		Potassium (mg/day) *		Sodium:Potassium Molar Ratio *		Creatinine (mg/day)
	Mean(sd)	95% CI	Mean(sd)	95% CI	Mean(sd)	95% CI	
**Men:**							
18–24 years	3866 (1554)	3405, 4327	2766 (813)	2525, 3008	2.5 (1.1)	2.2, 2.9	1706 (431)
25–44 years	3795 (1503)	3372, 7217	3058 (957)	2786, 3329	2.2 (0.8)	2.0, 2.4	1703 (482)
45–64 years	3931 (1520)	3517, 4347	3085 (1050)	2798, 3371	2.3 (1.0)	2.1, 2.6	1719 (401)
Total men	3865 (1515)	3618, 4113	2979 (955)	2822, 3135	2.4 (1.0)	2.2, 2.5	1710 (436)
Weighted mean ^†^	3854	3597, 4111	3005	2837, 3174	2.3	2.2, 2.4	N/A
**Women:**							
18–24 years	3017 (954)	2724, 3311	2193 (632)	1999, 2387	2.4 (0.8)	2.2, 2.7	1174 (232)
25–44 years	3035 (1241)	2706, 3365	2361 (839)	2138, 2583	2.3 (0.9)	2.1, 2.6	1188 (283)
45–64 years	2780 (1401)	2418, 3142	2751 (784)	2549, 2954	1.8 (0.7)	1.6, 2.0	1100 (350)
Total women	2934 (1235)	2738, 3130	2463 (798)	2337, 2590	2.1 (0.86)	2.0, 2.3	1151 (299)
Weighted mean ^†^	2926	2716, 3137	2497	2363, 2631	2.1	2.0, 2.2	N/A
Total men and women	3386 (1452)	3221, 3551	2712 (913)	2608, 2816	2.2 (0.93)	2.1, 2.3	1422 (465)

* excluding one outlier with potassium excretion of 10,605 mg/day; ^†^ weighted to reflect age and sex structure of the New Zealand population aged 18–64 years in 2012.
